# Non-Invasive Evaluation of Different Soil Tillage and Seed Treatment Effects on the Microbial Originating Physiological Reactions of Developing Juvenile Maize

**DOI:** 10.3390/plants11192506

**Published:** 2022-09-26

**Authors:** Antal Binder, Ildikó Jócsák, Zsolt Varga, Bence Knolmajer, Sándor Keszthelyi

**Affiliations:** 1 Department of Agronomy, Institute of Agronomy, Hungarian University of Agriculture and Life Sciences, Kaposvár Campus, Guba Sándor Street 40, H-7400 Kaposvár, Hungary; 2 Plant-Treat Ltd., Ady Endre Str. 12, H-8900 Zalaegerszeg, Hungary; 3 Institute of Plant Protection, Hungarian University of Agriculture and Life Sciences, Georgikon Campus, Deák Ferenc Str. 16, H-8360 Keszthelyi, Hungary

**Keywords:** juvenile stage, maize, non-invasive imaging, phytopathogenic agents, plant physiological stress, soil cultivation

## Abstract

The successful production of maize is fundamentally determined by a good choice of tillage type. Options include conventional tillage based on soil rotation, as well as a more recent conservation approach. Our aims were to determine the stress physiological effects of the plant remains left behind by different tillage procedures on the juvenile maize plants, combined with the effects of fungicide treatment on the seeds. These effects were followed and investigated by means of biophoton emission measurement, an in vivo and non-invasive imaging technique, along with chlorophyll content estimation, as well as microbial- and polymerase chain reaction-based identification of fungi presence. Our results confirmed the response reactions of maize triggered by a soil covering plant remains on the initial development and physiological involvement of maize. The positive effects of seed treatment on initial development are manifested only at the final stage of the experiment. The fungal microbiological analysis confirmed the dominant presence of necrotrophic parasites on plant residues, the stress-inducing properties of which were possible to monitor by biophoton emission. Furthermore, the presence of *Fusarium* spp. was confirmed by PCR analysis from samples treated with plant residues.

## 1. Introduction

Maize is one of the most important cultivated plants worldwide, which is confirmed by an annual cultivation area of more than 197 million hectares and an annual harvest of 1.125 million tonnes of grain in 2021 [1]. Its successful production is determined by a number of agro-technical, tillage, and plant protection technological interventions [2]. The fundamental element of this complex is the good choice of the type of tillage. 

There is so-called conventional tillage based on soil turning and residue-burying [3], as well as the recently introduced conservation approach without soil rotation, which leaves crop residues on the surface [4]. In recent decades’, emphasis in soil tillage has, in addition to increasing crop productivity, included the concept of conservation tillage (from minimum to zero tillage technologies) that ensures reduction in soil erosion and water preservation through maintenance of the soil surface covered by plant residue [5]. Conservation tillage, including no-tillage soil cultivation, organic mulch soil cover, and crop species diversification, was practised globally on about 200 M ha (on 14% of field cropland), in all continents in 2021 [6]. There are many advantages and disadvantages of conservation tillage in contrast to conventional tillage. Its main benefits are less soil erosion, moisture evaporation, and compaction, which result in more fertile and resilient soils due to organic carbon retention. Conversely, the main disadvantage is that gullies can form in the fields, which originates from special soil use. In addition, the number of herbicide applications can increase due to the lack of some soil rotation techniques [7]. This change constitutes an increase of 27% globally since 2013 [8]. In the last 8 years, conservation agriculture cropland has expanded at an average rate of more than 5.3 M ha per year. Over the last decade, the adoption of this practise has been intense mainly in Europe and Africa [6].

Conservation tillage could be important in areas where water is a major limitation to production, and where soil structure is poor and erosion is a problem [5]. The aim of these new soil tillage procedures will be long-term protection of the resources of the soil, reduced machinery usage, and savings in fuel and labour costs [4]. However, mulching combined with no-tillage can be an important component in combating drought stress in a simple, cost-efficient and easy-to-implement way, thus providing significant support in drought-affected or arid regions [9]. In addition, several phytosanitary concerns can emerge in the post-crop stock, which is originated from the surface left plant remains [10] as opposed to conventional tillage, where plant remains are rotated into the soil, thus preventing the transmissions of infections. These plant protection problems may be particularly important in cereals or maize bi- or monocultures [9]. Saprotrophic and soil-dwelling phytopathogens (*Fusarium* spp., *Nigrospora oryzae*) are of particular interest in these cases, which are able to infest the developing maize plants from the surface cereal remains left in the soil during the whole vegetation cycle [11]. Naturally, these serious production risks need to be taken into account in the case of conservation tillage application. 

Molecular biological-based detections of stress processions triggered by different phytopathogens are widely known and applied methods in maize pathological research [12,13]. On the contrary, non-invasive imaging of phytopathological reactions has been less employed by the scientific practice in plant disciplines as of yet [14]. One such method is the detection of biophoton emission (BPE) and its visual displaying. Due to its ultra-weak intensity, spontaneous chemiluminescence is invisible to the naked eye [15], and only possible to detect via a highly sensitive charge-coupled device (CCD) camera. Ultra-weak biophoton emission arises in response to biotic and abiotic stress, and is surmised to stem from the endogenous production of metastable excited states, as a result of spontaneous photon emission ”reflecting” the oxidation status of the organism [16]. The technique is suitable for the displaying of physiological disorders triggered by most different biotic or abiotic stressors in a non-destructive way [17,18].

Our aims were to determine the stress physiological effects of the plant remains left behind by different tillage procedures on juvenile maize plants. We intended to reveal to what extent the stress in the developing plants is triggered by phytopathogenic agents originating from plant remains and whether these physiological can be visualized by means of biophoton emission. In addition, we wanted to justify the presence of the most significant microorganism that has infested the plant with the help of a molecular biological assay. Eventually, we aimed to draw conclusions about the phytosanitary consequences of different soil tillage systems by means of the achieved information.

## 2. Results

### 2.1. Plant Growth and Chlorophyll Content Estimation

**Figure 1:**
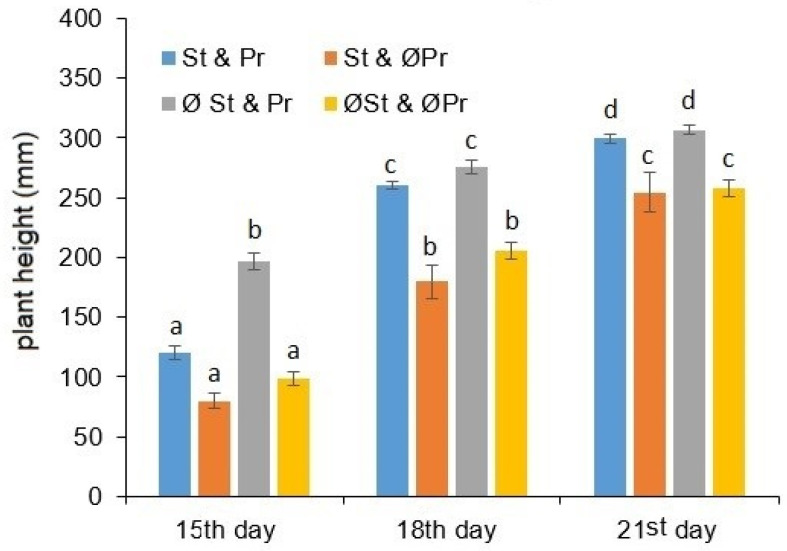
Plant height tendencies as a function of exposure time, as well as seed treatment and the presence of plant remains. St & Pr: seeds treated by fungicides combined with plant remains left on the soil surface; St & ØPr: seeds treated by fungicides without plant remains; ØSt & Pr: seeds without fungicide treatment combined with plant remains left on the soil surface; ØSt & ØPr: seeds without both fungicide treatment and plant remains. a, b, c, d: small letters indicate significant difference (*p* ≤ 0.05) between means of different exposure times.

The measured plant heights as a function of the exposure times are presented in Figure 1. The effects of different treatments on the growth of the juvenile stage maize have unequivocally been proven by the statistical analysis. The plant height was significantly influenced by the fact of seed treatment and the presence of plant remains (df = 3; *p* = 9.05 × 10^−17^). It can be ascertained that the seed germinated among plant remains are better developed than the plants situated in the clear soil surface both in the case of samples with treatments (df = 1; *p* = 0.021) or without (df = 1; *p* = 0.036) seed. The height of those plants that germinated from fungicide-treated maize seeds did not exceed the height of the plants developed without seed treatment. The difference originating from this plant protectant treatment only showed up at the end of the experimental analysis, on the 21st day.

Overall, the consequences of the seed treatment on plant height were not confirmed by statistical assays of both the treatments with (df = 1; *p* = 0.062) and without (df = 1; *p* = 0.955) plant residues.

**Figure 2:**
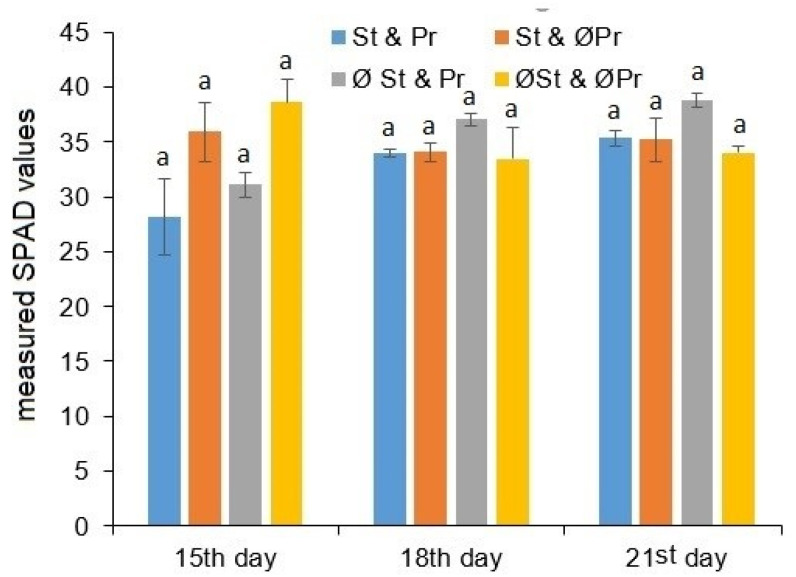
Chlorophyll content estimation (Soil Plant Analysis Development, SPAD index) as a function of exposure time, as well as seed treatment and the presence of plant remains. St & Pr: seeds treated by fungicides combined with plant remains left on the soil surface; St & ØPr: seeds treated by fungicides without plant remains; ØSt & Pr: seeds without fungicide treatment combined with plant remains left on the soil surface; ØSt & ØPr: seeds without both fungicide treatment and plant remains. a: small letter indicates significant difference (*p* ≤ 0.05) between means of different exposure times.

The results of the chlorophyll content estimation are shown in Figure 2. No differences were seen during the first evaluation, and the effects of the seed treated by fungicide and the presence or lack of dead plant remains on the changing of chlorophyll content could not be statistically confirmed (*p* > 0.05).

### 2.2. Imaging of Biophoton Emission

**Figure 3:**
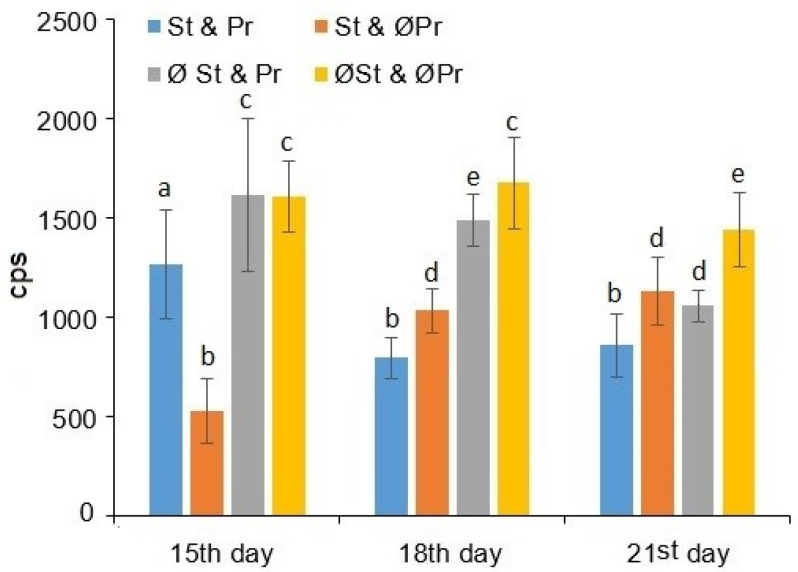
Sum of overall count per second (cps) values of biophoton emission (BPE) as a function of exposure time, as well as seed treatment and the presence of plant remains. St & Pr: seeds treated by fungicides combined with plant remains left on the soil surface; St & ØPr: seeds treated by fungicides without plant remains; ØSt & Pr: seeds without fungicide treatment combined with plant remains left on the soil surface; ØSt & ØPr: seeds without both fungicide treatment and plant remains. a, b, c, d, e: small letters indicate significant difference (*p* ≤ 0.05) between means of different exposure times.

The cps values of BPE can be seen in Figure 3. It has been confirmed by the two-way ANOVA that both the exposure time (df = 3; *p* = 0.0025), the different treatments together (df = 3; *p* = 0.0039), and their interaction (df = 6; *p* = 0.0062) have a significant relationship with the measured values of ultra-weak photon emission. The effects of seed treatment on the values of ultra-weak photon emission are uniformly statistically verifiable both in the case of the presence (df = 1; *p* = 0.0165) and the absence (df = 1; *p* = 0.0037) of plant residues. In contrast, the same comparison of the plant residues was not statistically verifiable.

**Figure 4:**
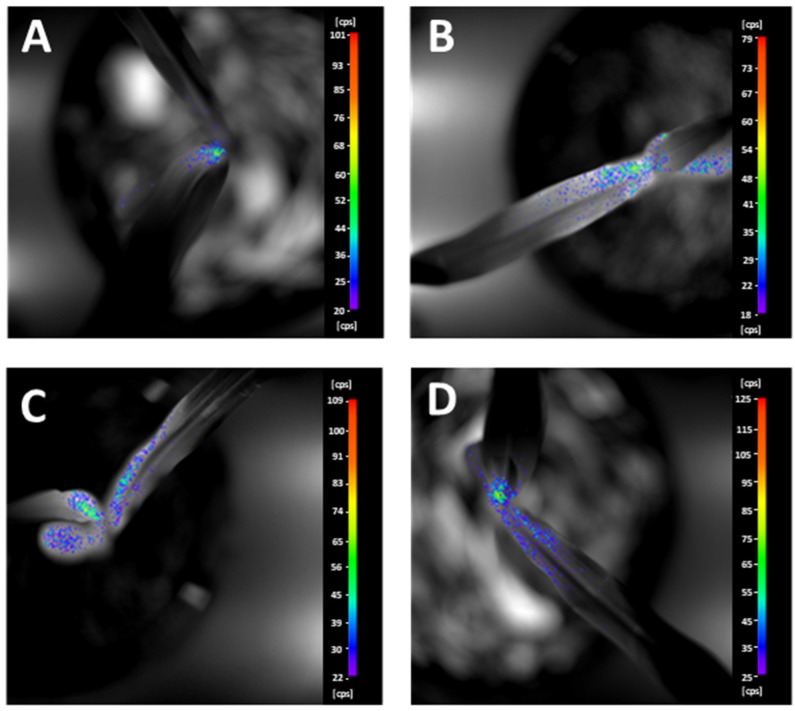
Images of biophoton emission (BPE) at the last sampling day. (**A**) St & Pr: seeds treated by fungicides combined with plant remains left on the soil surface; (**B**) St & ØPr: seeds treated with fungicides without plant remains; (**C**) ØSt & Pr: seeds without fungicide treatment combined with plant remains left on the soil surface; (**D**) ØSt & ØPr: seeds without both fungicide treatment and plant remains.

The images of the BPE measurement are presented in Figure 4. The pseudocolour intensities followed the same pattern that was already observed in Figure 3. According to the pseudocolors generated by the IndiGo™ 2.0.5.0. software (Berthold Technologies, Bad Wildbad, Germany) of NightShade LB 985 in-vivo Plant Imaging System, the highest BPE values were detected from those plants that were not treated at all (neither with plant residues, nor fungicide pretreatment), whereas the lowest level of signals originated from those that were treated with fungicide and plant residues at the same time.

### 2.3. Microfungi Assortments Deriving from Plant Remains

Table 1 contains the list of the observed microfungi species and their infestation frequency related to the examined plant and seed remains.

A total of 21 fungi species were determined from the plant remains originating from maize, belonging to 17 genera. The dominant genera were *Alternaria* and *Cladosporium*. From the soil-dwelling fungi, the most frequent were *Fusarium* and *Penicillium*, but *Aspergillus, Trichoderma, Rhizopus*, and *Mucor* could be also detected in more cases. The phytopathogenic species deriving from leaf remains were *Bipolaris maydis, Puccinia sorghi*, and *Phoma* spp., while *Nigrospora oryzae* could be detected from the inner tissues of the maize stalks. The most dominant species was *Rhizopus stolonifer* over the course of seed surface examinations, which was unequivocally proven by the 90% germination and the 100% frequency.

*Fusarium* species were one of the most important phytopathogen agents from the maize remains originating from the stubble. In the most numerous cases, *Fusarium* species were detectable from the surface of seeds originating from the stubble, followed by their detection from leaf and husk remains. According to the morphological features, the dominant *Fusarium* species was *F. verticilliodes*, forming microconidia.

### 2.4. Confirmation of the Microfungi Infestation

**Figure 5:**
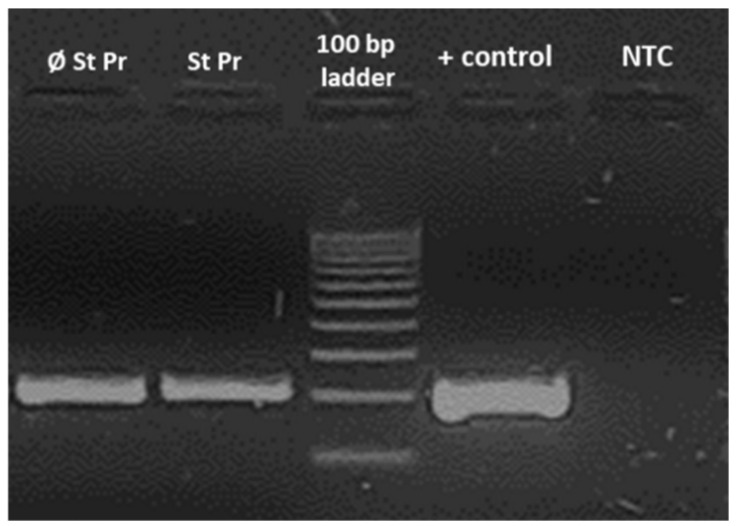
Identification of *Fusarium* spp. by means of PCR reaction.

In order to test our hypothesis on the fungal origin of the experienced stress, the samples with plant residues were tested against the presence of *Fusarium* species, the results of which are shown in Figure 5.

According to the results of the PCR test (Figure 5), both groups of samples treated with plant residues were infected by *Fusarium*. However, the intensity of the bands indicates a more intensive infestation in those samples that were not subjected to fungicide seed pretreatment, since the same amount of DNA (20 ng) was loaded on each well of the gel. In the case of the non-template control (NTC), the PCR amplification did not result in detectable amplicon production compared to the positive control and the infected samples (Figure 5). 

## 3. Discussion

Our results confirmed the response reactions of maize triggered by soil covering including plant remains on the initial development and physiological involvement of maize.

It has been proven that the mulching effect of stem cuttings plays an important role in water retention, which was reflected in the more vigorous initial growth of mulched plants [9,19,20]. Our investigations pointed out, similarly to earlier works of Ghosh et al. (2006), Ali et al. (2018), and Akhtar et al. (2019) [9,19,20], that the shading effect of plant residues helps to maintain soil moisture and optimal soil temperature for the germination and the development of maize, which results in the advancement of the initial developmental vigour of maize and other emerging crops. This finding was confirmed by our results, indicating that mulching has a positive effect in increasing soil water retention, which therefore is a promising option to counteract drought stress. The explanation of this phenomenon is rather complex, because the rapid decomposition will provide a boost of nutrients during the period of crop growth [21]. Nevertheless, the impact on soil physical conditions are significant [22]. In contrast, there are some scientific works which published about an opposite effect on the developing plants. According to the results based on the bioassays of Bradow and Connick (1990) [23], (E)-2-hexenal, nonanal, 3-methylbutanal, and ethyl 2-methylbutyrate are such inhibitory volatiles. All the volatile mixtures originating from plant residues examined contained at least one compound that greatly inhibited seed germination.

The positive effects of seed treatment on initial plant development—which can be observed in the plant height—are manifested only at the final stage of the experiment. In the period of this initial development, other agrotechnical factors have a very decisive effect on plant development [24]. The fungal microbiological analysis confirmed the dominant presence of necrotrophic parasites on plant residues. This observation is proven by scientific studies [25,26]. Overall, the presence of these phytopathogen fungal species in the immediate vicinity of emerging maize can pose a fundamental risk to cultivated cereals’ health.

BPE provides information about the oxidative status of the plant tissues [27,28,29] due to the connection between ultra-weak bioluminescence and reactive oxygen species: when the generated excited intermediaries return into their ground state, photons are released. According to our results, starting from the 18th day, it was statistically proven that seed treatments combined with plant residues resulted in lower BPE values, indicating a lower level of lipid oxidation, and thus lower stress status. It was possible to observe the same phenomenon in the case of all the treatments, which had higher BPE values than seed treatments combined with plant residues. 

Naturally, there are several biotic and abiotic stress factors that play a determinative role in the healthy initial development of maize or other field crops. An important one among these factors is the choice of a good tillage system that enables soil moisture preservation, overcoming infectious agents or maintaining diverse biological and soil life. Therefore, as a continuation of our research, it will be worthwhile to investigate the effectiveness of stem residue mulching in overcoming drought stress.

According to our results, the proper choice of these factors, or, in other words, a combination of abiotic and biotic elements, may contribute to the increase of qualitative and quantitative values of field crop yield. The detection and objective measurement of the potential stress phenomena in plants is an important part of this complex, because it may only be possible to recognise hidden physiological processes via the application of non-invasive in vivo analytical techniques.

## 4. Materials and Methods

### 4.1. Sampling and Experimental Settings

In order to evaluate the stress physiological response of the juvenile maize triggered by phytopathogenic agents originating from the plant remains, healthy, contamination-free maize was sown in plastic pots (1 seed/pot) on 5 November 2021. The seed was a commercial Syngenta hybrid, which belongs to the maturity group FAO 380. These experimental plant materials were placed in a climate chamber under the climatic parameters mimicking favourable spring weather for maize (photoperiod: 16L:8D; in the diurnal stage: 23 °C, 60% rh; in the nocturnal stage: 10 °C, 30% rh). Four treatments in five repetitions were set up:

1. Seeds treated by fungicides combined with plant remains left on the soil surface (St & Pr);
2. Seeds treated by fungicides without plant remains (St & ØPr);
3. Seeds without fungicide treatment combined with plant remains left on the soil surface (ØSt & Pr);
4. Seeds without both fungicide treatment and plant remains (ØSt & ØPr).

The applied fungicide active ingredients for the seed treatment were 37.5 g L^−1^ fludioxonil, 300 g L^−1^ thiabendazol, 29 g L^−1^ metalaxyl-M, and 15 g L^−1^ azoxystrobin (dose: 8.5 mL/50,000 seed). The plant remains were obtained from the soil surface of the harvested maize field of that year, which contained mixed stalk, leaf, and cob maize remains. The plant remains were collected from the harvested maize stable on 5 November 2021. These dead plant remains were placed in the pot so that it completely covered the soil surface (dose: 20–30 g/dm^2^) at the same time as sowing, and was not removed until the end of the experiment.

This plant material was subjected to phytopathological examination.

Traditional (plant height, chlorophyll content estimation based on SPAD) and non-invasive stress (detection of biophoton emission) analytical assays were carried out on the 15th, 18th, and 21st days after sowing. Between the analytical recordings, the plants were put back into the climate chamber.

### 4.2. Non-Invasive Imaging of Plant Stress by Means of Detection of Biophoton Emission

#### 4.2.1. Chlorophyll Content Estimation (SPAD Index) Measurement

SPAD index, as a non-invasive measurement for chlorophyll content estimation, was measured by reading five individual points on each plant of each treatment using SPAD (Soil Plant Analysis Development–SPAD-502; Konica Minolta Sensing Inc., Beijing, China) equipment. 

#### 4.2.2. Biophoton Emission (BPE) Measurement

For measuring BPE, the NightShade LB 985 in vivo Plant Imaging System (Berthold Technologies GmbH & Co.KG, 75323 Bad Wildbad, Germany), equipped with a sensitive, thermoelectrically cooled slow-scan NighOwlcam CCD device, was employed. The instrument was controlled by the IndiGo™ 2.0.5.0. software. Intensities of light were converted into counts per second (cps) by using the controlling software. The exposure time was kept at 60 s using a pixel binning of 4 × 4. In the duration of taking the images, both the “background correction” and the “cosmic suppression” options were enabled to ensure the elimination of high intensity pixels potentially caused by cosmic radiation. One pot for each treatment of the seedlings to be imaged was placed in the dark imaging box for 30 min in order to have dark adaptation, after which luminescence data were acquired for 10 min.

### 4.3. Microbial Cultivation of Potential Pathogens from the Plant Remains

As a prelude to the laboratory work, the maize plant residues were first grouped for microbial identification as follows:

1. leaf remains (any part of dry and damaged leaves);
2. maize stalks (longitudinal sections);
3. husk remains;
4. maize seeds and ear remains left on stubble;
5. sown maize seeds.

These plant materials were cut into 10–15-centimetre pieces. In addition to surface cleaning, we carried out disinfection with the aim of more accurate mapping of the microfungi spectrum that occurred on these plant remains.

We placed 4 pieces in 4 replicates in Petri-dishes from this prepared plant material, and these were put into a wet chamber. After this, we put plastic mosquito nets on the wet filter papers in order to prevent the samples from wetting. This ensured the optimal conditions of microfungi development and allowed the evaluation of the samples. 

The examinations were carried out under natural illumination and at 21 ± 2 °C. The incubation time of plant remains was 48 h. The identification of the potential microfungi was carried out with classic mycological methods. The primary evaluation was implemented by binocular stereomicroscope (at 50× magnification); then, we made the preparation of microfungi for light microscopy (between 100–1000× magnification), during which the potential fungal agents were identified based on their morphological property and sizes.

In addition to the vegetative plant remains, we put 4 × 25 maize seeds on the wet filter paper. The determination of microbial agents found on the seeds was started after 7 days of incubation.

### 4.4. Polymerase Chain Reaction Assay 

The primers of the PCR reaction were the following: Fps-F: 5′ CGCACGTATAGATGGACAAG 3′, as described in Jurado et al. (2005) [30], and Fus-R 5′ GGCGAAGGACGGCTTAC 3′) [31]. The size of the amplicon was 200 bp.

The PCR reaction was set up to 25 µL using 5xPCR master mix (PCRBIO Taq Mix; PCR Biosystems Ltd. Aztec House 397-405 Archway Road London, N6 4ER, UK), 20 pmol of each primer, and 20 ng of the extracted DNA.

The PCR reaction was completed in a GeneAmp^®^ PCR System 2700 thermal cycler (Lingley House 120, Birchwood Boulevard, Warrington, Cheshire, WA3 7QH, UK) with the following parameters: initial denaturation at 94 °C for 85 s, 25 cycles of 95 °C for 35 s, 67 °C for 30 s, and 72 °C for 30 s, and a final extension step at 72 °C for 5 min.

The PCR products were electrophoresed in a 2% Tris-acetate-EDTA–agarose gel and stained with SYBR™ Gold Nucleic Acid Gel Stain (Thermo Scientific™ 840274200, 168 3rd Ave, Waltham, MA, USA); the bands were visualised under UV light using a TCX 20 M transilluminator (EuroClone S.p.A. Via Figino, 20/22-20016, Pero, MI, Italy). Fragment sizes were determined with reference to a 100 bp DNA ladder: GeneRuler™ 100 bp DNA Ladder (Thermo Scientific™ 840274200, 168 3rd Ave, Waltam, MA, USA).

### 4.5. Statistical Analysis

The presented values were plotted as an average of five independent measurements and presented together with standard errors (± SD). The effect of the presence or lack of plant residue imitating the different tillage systems and seed treatment on the measured variables (plant height, chlorophyll content estimation, biophoton emission) was determined by two-way ANOVA (*p* ≤ 0.05) and the Tukey test, using the SPSS 20.0 statistical program.

## Figures and Tables

**Table 1:** Fungi species/genera isolated from the seed surface and the maize remains originating from the stubble. Infestation/occurrence frequencies (%) of the species are represented by the numbers.

Genera	Species	Plant Remains				Seed
		Leaf	Stalk	Husk	Ear	
*Alternaria*	*A. alternata*	100	45	6	-	-
	*A.* sp.					
*Cladosporium*	*C. herbarum*	95	80	95	1	-
	*C. cladosporioides*					
*Rhizopus*	*R. stolonifer*	-	20	-	21	100
*Fusarium*	*F. verticillioides*	25	15	25	38	1
	*F.* spp.					
*Epicoccum*	*E. nigrum*	30	6	6	-	-
*Gonatobotrys*	*G. flava*	30	-	12	-	-
*Acremoniella*	*A. atra*	35	25	-	1	-
*Pithomyces*	*P. chartarum*	6	-	-	-	-
*Penicillium*	*P.* spp.	-	6	25	64	4
*Trichoderma*	*T.* sp.	-	-	-	1	-
*Verticillium*	*V.* sp.	-	6	-	-	-
*Aspergillus*	*A. flavus*	-	12	-	-	-
	*A. clavatus*	-	-	-	-	1
*Mucor*	*M. mucedo*	-	6	-	-	-
*Nigrospora*	*N. oryzae*	-	31	-	-	-
*Phoma*	*Ph.* sp.	12	-	-	-	-
*Puccinia*	*P. sorghi*	12	-	-	-	-
*Bipolaris*	*B. maydis*	12	-	-	-	-
